# Beyond prevalence: significance and differential impact of echocardiographic abnormalities in dialysis patients

**DOI:** 10.1007/s40620-024-01963-2

**Published:** 2024-06-04

**Authors:** Chih-Hsueh Tseng, Yi-An Hu, Yung-Tai Chen, Wen-Chung Yu, Chih-Ching Lin, Szu-Yuan Li

**Affiliations:** 1 Division of Cardiology, Department of Medicine, Taipei Veterans General Hospital, Taipei, Taiwan; 2 Division of Holistic and Multidisciplinary Medicine, Department of Medicine, Taipei Veterans General Hospital, Taipei, Taiwan; 3 Institute of Emergency and Critical Care Medicine, National Yang-Ming Chiao-Tung University, Taipei, Taiwan; 4 Division of Nephrology, Department of Internal Medicine, Taipei Veterans General Hospital, Taipei, Taiwan; 5 Institute of Clinical Medicine, School of Medicine, National Yang Ming Chiao Tung University, Taipei, Taiwan; 6 Department of Medicine, Taipei City Hospital Heping Fuyou Branch, Taipei, Taiwan; 7 School of Medicine, National Yang-Ming Chiao-Tung University, No. 201, Sec. 2, Shih-Pai Road, Taipei, 112 Taiwan

**Keywords:** End-stage kidney disease, Echocardiogram, Hemodialysis, Peritoneal dialysis, Heart failure, Systolic dysfunction

## Abstract

**Background:**

Echocardiography is commonly used to assess hydratation status and cardiac function in kidney failure patients, but the impact of structural or functional abnormalities on the prognosis of kidney failure patients was yet to be investigated. This study aimed to investigate the prevalence and clinical significance of echocardiographic abnormalities in kidney failure patients.

**Methods:**

This study included 857 kidney failure patients who underwent echocardiography at dialysis initiation. Patients were followed up for a median of 4.2 years for the occurrence of major adverse cardiovascular events (MACE) and all-cause mortality.

**Results:**

Among the 857 patients studied, 77% exhibited at least one echocardiographic abnormality. The most common abnormalities were left ventricular hypertrophy and left atrial enlargement, but they were not significantly correlated with poor outcomes. Instead, the primary predictors of both major adverse cardiovascular events and mortality in kidney failure patients were left ventricular systolic function, right ventricular systolic function, left ventricular volume index, and valvular abnormalities. Although diastolic dysfunction was linked to major adverse cardiovascular events, it was not associated with mortality. Furthermore, the study revealed that increased left ventricular volume index and left ventricular systolic dysfunction had a more significant impact on peritoneal dialysis (PD) patients than on hemodialysis (HD) patients.

**Conclusion:**

This study provides insights into the echocardiographic abnormalities and their association with adverse outcomes in kidney failure patients, which can help clinicians optimize the management of patients and closely monitor possible high-risk populations.

**Graphical abstract:**

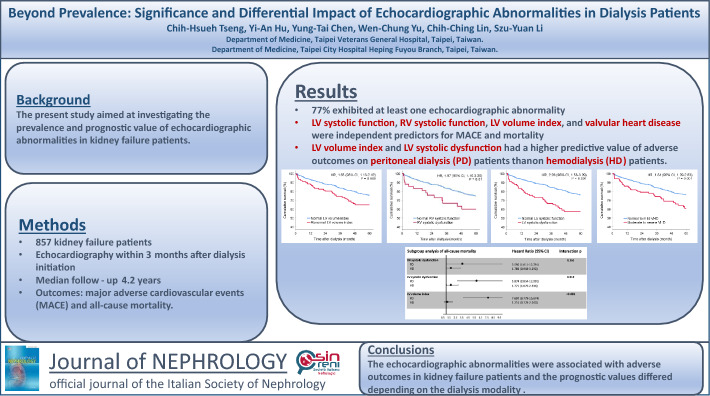

**Supplementary Information:**

The online version contains supplementary material available at 10.1007/s40620-024-01963-2.

## Introduction

Kidney failure patients have a high mortality rate, with cardiovascular disease being the leading cause [1]. The kidneys have an essential role in maintaining blood pressure and eliminating waste products and surplus fluids from the body. However, when the kidneys fail to function properly, waste products and fluids can accumulate in the body, leading to type 4 cardiorenal syndrome and cardiac remodeling [2]. This process involves structural and functional changes in the heart, primarily left ventricular dilatation, hypertrophy, and diastolic dysfunction, which significantly increase the risk of cardiovascular events [3]. In addition, accelerated atherosclerosis, dialysis-related hemodynamic instability, and acute electrolyte changes, all contribute to the high cardiovascular mortality in kidney failure patients [4, 5]. The current heart failure classification systems, such as the AHA and NYHA systems, are largely based on symptoms and functional capacity. However, these systems may not be suitable for patients with kidney failure due to the complex interactions between fluid overload, left ventricular (LV) preload, and structural changes in the heart. Additionally, kidney failure patients may have different symptoms and functional limitations compared to patients with heart failure alone.

Echocardiography is commonly used as the primary method for evaluating cardiac function and structure. As a result, it is also considered one of the most accessible and robust examinations for evaluating heart failure in patients with kidney failure [6–8]. According to the Kidney Disease Outcomes Quality Initiative (KDOQI) guideline recommendation, all new dialysis patients should undergo an echocardiographic exam in the initial phase of dialysis once the patient has achieved dry weight to access the fluid status, and every 3 years thereafter [9]. However, not all dialysis centers have access to echocardiography or trained personnel to perform and interpret the exams. There is also a lack of standardized protocols for echocardiography in kidney failure patients, in addition, the cost of echocardiography can be a barrier for some patients or healthcare systems.

When using echocardiography to evaluate kidney failure patients, several parameters are examined to assess cardiac function. Although left ventricular ejection fraction (LVEF) is commonly considered a crucial parameter, other factors such as left atrial size, left ventricular mass, and diastolic function may also hold significance. However, the impact of these echocardiographic abnormalities on the predictive value of adverse cardiovascular outcomes in kidney failure patients is yet to be investigated. The present study aims at investigating the potential clinical implications of baseline echocardiography and evaluating the predictive value of echocardiographic parameters on the outcomes.

## Methods

### Study design and patient selection

We included patients who started long-term outpatient peritoneal dialysis (PD) (*n* = 383) or chronic hemodialysis (HD) (*n* = 664) between 2006 and 2020 at Taipei Veterans General Hospital and excluded those who did not undergo echocardiogram examination within 3 months of starting dialysis (*n* = 190). The primary outcomes of this study were major adverse cardiovascular events (MACE) and all-cause mortality; patients were followed up for 5 years or until the end of the study (July 31, 2021). The baseline characteristics of the patients, such as age, gender, height, body weight, dialysis modality, comorbidities, cause of kidney failure, and medication history were collected from electronic medical records. The investigation adhered to the Declaration of Helsinki with the approval of the Institutional review board of Taipei Veteran General Hospital.

### Echocardiography parameters and definition of abnormality

Left ventricular ejection fraction was obtained manually at end-diastole and end-systole in four-chamber and two-chamber views using the modified biplane Simpson’s method [10]. Left ventricular systolic dysfunction was defined as a left ventricular ejection fraction of 45% or lower. Cardiac abnormality was defined according to the American Society of Echocardiography (ASE) and European Society of Cardiology guidelines with minor modifications to account for kidney failure patients [11–13]. For the purpose of this study, seven parameters were utilized to assess cardiac function. Left ventricular hypertrophy (LVH) was defined as left ventricular mass index (LVMi) greater than 100 g/m^2^ for women and greater than 130 g/m^2^ for men. Increased left ventricular volume index (LVVi) was defined as greater than 86 ml/m^2^ end-diastolic volume or greater than 37 ml/m^2^ end-systolic volume. Right ventricular systolic dysfunction was defined as tricuspid annular plane systolic excursion (TAPSE) < 1.6 cm or right ventricular pulsed doppler peak systolic velocity at the annulus (RVS’) < 10 cm/s [14]. Pulmonary hypertension was defined as pulmonary artery systolic pressure more than 35 mmHg. Left atrial enlargement (LAE) was defined as left atrial (LA) size greater than 40 mm. Diastolic dysfunction was defined according to the 2016 ASE guideline recommendation but using left atrial size as a surrogate of the left atrial volume index [15]. Valvular diseases with moderate to severe stenosis or regurgitation were also considered as a distinct parameter of cardiac abnormality.

### Outcomes of interest

Patients were followed up for all-cause mortality and major adverse cardiovascular events including non-fatal stroke, non-fatal myocardial infarction, and hospitalization for heart failure.

### Statistical analysis

Baseline characteristics were presented as means with standard deviation for continuous variables, and percentages for categorical variables. Comparisons of proportions between groups were made using the Chi-square test. Continuous variables were compared using independent *t* test. Event rates were presented with Kaplan–Meier methods and compared by log-rank test. Cox proportional hazard model was utilized to access the risk of each echocardiographic abnormality on major adverse cardiovascular events and mortality. Multivariate model was based on the factors with *p* < 0.1 in the univariate analysis. All analyses were performed using IBM SPSS Statistics 25.0 software (Armonk, NY: IBM Corp.). All the tests performed were 2 sided, and *p* value < 0.05 was considered statistically significant.

## Results

### Study cohort

Between January 2006 and December 2020, a total of 1047 patients began long-term dialysis treatment in Taipei Veterans General Hospital, with 383 receiving PD and 664 receiving HD. After excluding 190 patients who did not receive an echocardiogram within 3 months of dialysis initiation, 857 patients met the study inclusion criteria. The mean age of the study population was 62 ± 12 years, with 445 (52%) patients being male, 275 (32%) receiving PD, and 131 (15%) having a history of heart failure. The most common causes of kidney failure were diabetes mellitus (39%), hypertension (20%), and glomerulonephritis (14%).

Among the study population, 659 patients (77%) had one or more echocardiographic abnormalities. Patients with diabetes, a history of heart failure, and higher body weight were more likely to have abnormal echocardiograms (Table 1).

**Table 1:** Baseline Characteristics of the Study Populations

	Without echocardiographic abnormalities (*n* = 198)	With echocardiographic abnormalities (*n* = 659)	*p* value
Demographics			
Age, years	59.87 ± 16.51	62.33 ± 16.65	0.068
Female sex, *n* (%)	89 (45%)	323 (49%)	0.316
Height, cm	162.38 ± 8.76	161.44 ± 8.63	0.184
Body weight, kg	60.19 ± 12.77	63.29 ± 14.76	0.007
Medical history, *n* (%)			
Hypertension	149 (75%)	545 (83%)	0.029
Diabetes mellitus	69 (35%)	321 (49%)	0.005
Atrial fibrillation	72 (11%)	5 (3%)	< 0.001
Coronary artery disease	28 (14%)	132 (20%)	0.045
Congestive heart failure	15 (8%)	116 (18%)	< 0.001
Cerebrovascular accident	16 (8%)	43 (7%)	0.473
Causes of kidney failure, *n* (%)			
Hypertension	40 (20%)	134 (20%)	0.960
Diabetic kidney disease	63 (32%)	272 (41%)	0.014
Chronic glomerulonephritis	38 (20%)	80 (12%)	0.023
SLE	8 (4%)	39 (6%)	0.264
Polycystic kidney disease	8 (4%)	15 (2%)	0.246
Others*	41 (21%)	119 (18%)	0.416
Medication, *n* (%)			
ACEi or ARBs	75 (38%)	254 (39%)	0.866
Beta-blockers	73 (37%)	309 (47%)	0.012

*ACEi* angiotensin converting enzyme inhibitors, *ARBs* angiotensin receptor blockers, *SLE* systemic lupus erythematosus

*Including idiopathic cause or acute kidney injury of infection, toxin, medications, or hemodynamic change

### Echocardiographic findings in kidney failure patients

**Fig. 1:**
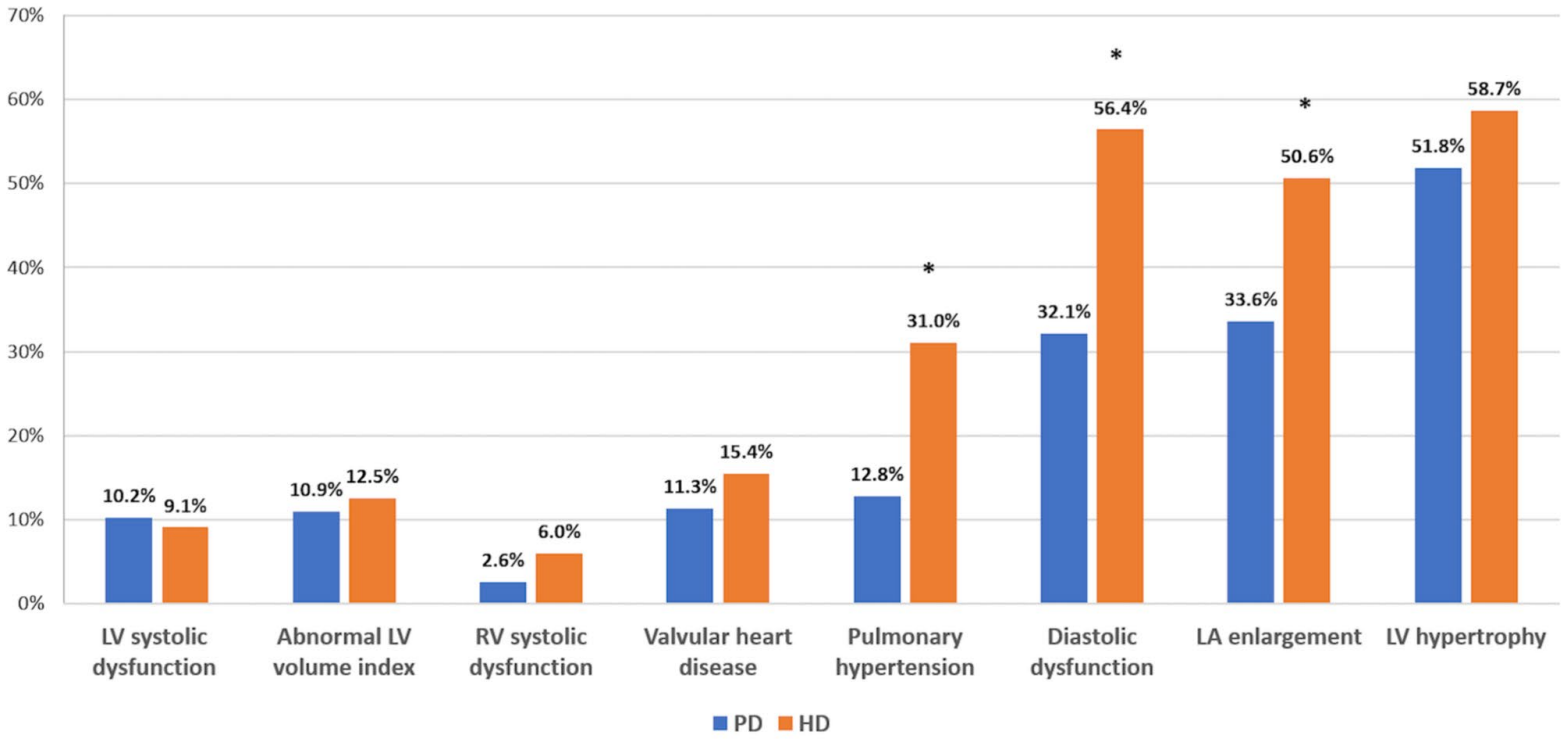
Prevalence of echocardiographic abnormalities in dialysis patients. *HD* hemodialysis, *PD* peritoneal dialysis. *p < 0.05

Most patients with an abnormal echocardiogram have more than one abnormal parameter. The most frequently observed abnormalities were left ventricular hypertrophy (56%), diastolic dysfunction (49%), and left atrial enlargement (45%). Mitral regurgitation was the most common valvular abnormality, present in 9% of patients. Right ventricular systolic dysfunction was observed in 5% of patients (Table 2). The average age was higher among the HD patients compared to PD patients (65.68 ± 16.07 vs 53.42 ± 14.67, *p* < 0.001). In addition, a greater prevalence of left atrial enlargement, diastolic dysfunction, and pulmonary hypertension among HD patients was also identified in the present study (Fig. 1). The echocardiographic findings relative to the type of dialysis vascular access are presented in Supplementary Table 1. Notably, left atrial size was significantly larger in subjects with arteriovenous fistulas (AVFs) compared to those using central venous catheters (CVCs). Apart from this distinction, other parameters including left ventricular mass index, left ventricular volume index, left ventricular ejection fraction, diastolic function, pulmonary artery systolic pressure (PASP), and right ventricular function showed no significant differences between the two groups.

**Table 2:** Baseline echocardiographic parameters of the study patients (*n* = 857)

Echocardiographic parameters	Values
LV mass	
LV hypertrophy	484 (56%)
LVMi, g/m^2^	125.03 ± 39.60
LV volume index	
LV volume diastolic index > 86 or systolic > 37 ml/m^2^	103 (12%)
LV volume diastolic index, ml/m^2^	51.00 ± 18.31
LV volume systolic index, ml/m^2^	23.33 ± 12.56
LVEF	
LVEF ≦ 45%	91 (11%)
Left atrium	
LA enlargement	387 (45%)
LA size, mm	40.3 ± 7.6
Diastolic function	
Diastolic dysfunction	417 (49%)
LA size > 40 mm	387 (45%)
TR peak velocity > 2.8 m/s	372 (43%)
Med e’ < 7 cm/s	697 (81%)
Med E/e’ > 15	433 (51%)
Valvular heart disease	
≧ moderate aortic/mitral valvular heart disease	121 (14%)
≧ moderate mitral stenosis or regurgitation present	82 (10%)
≧ moderate mitral stenosis	1 (0%)
≧ moderate mitral regurgitation	81 (9%)
≧ moderate aortic stenosis or regurgitation present	52 (6%)
≧ moderate aortic stenosis	15 (2%)
≧ moderate aortic regurgitation	38 (4%)
RV systolic function	
RV systolic dysfunction	42 (5%)
PASP, mmHg	35.6 ± 0.5
Pulmonary hypertension	372 (43%)

*PASP* pulmonary artery systolic pressure, *LA* left atrium, *LV* left ventricle, *LVEF* left ventricular ejection fraction, *LVMi* left ventricular mass index, *TR* tricuspid regurgitation

### Echocardiographic abnormalities and patient outcomes

During a mean follow-up period of 4.28 ± 2.61 years, a total of 183 patients developed major adverse cardiovascular events while 185 patients died. The cumulative survival rate at 1 year, 3 years, and 5 years was 93%, 84%, and 79%, respectively, which is consistent with the mortality rate of dialysis patients in Taiwan [16].

**Fig. 2:**
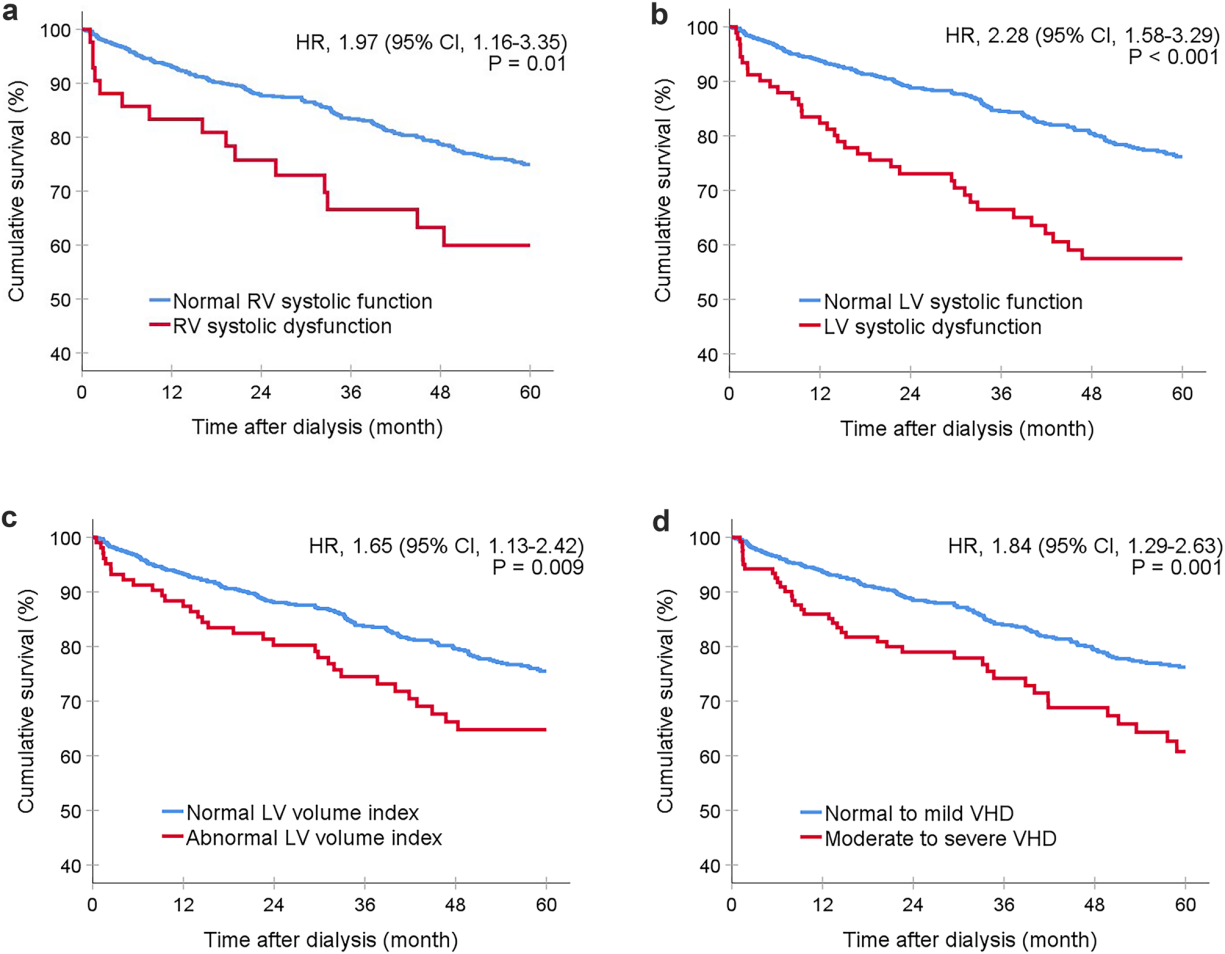
Survival analysis of abnormal echocardiographic parameters in the dialysis population. Kaplan–Meier survival curve analysis of mortality over a 5-year follow-up period in relation to specific echocardiographic abnormalities including right ventricular systolic dysfunction, left ventricular systolic dysfunction, left ventricular volume index, and moderate to severe valvular heart disease

**Fig. 3:**
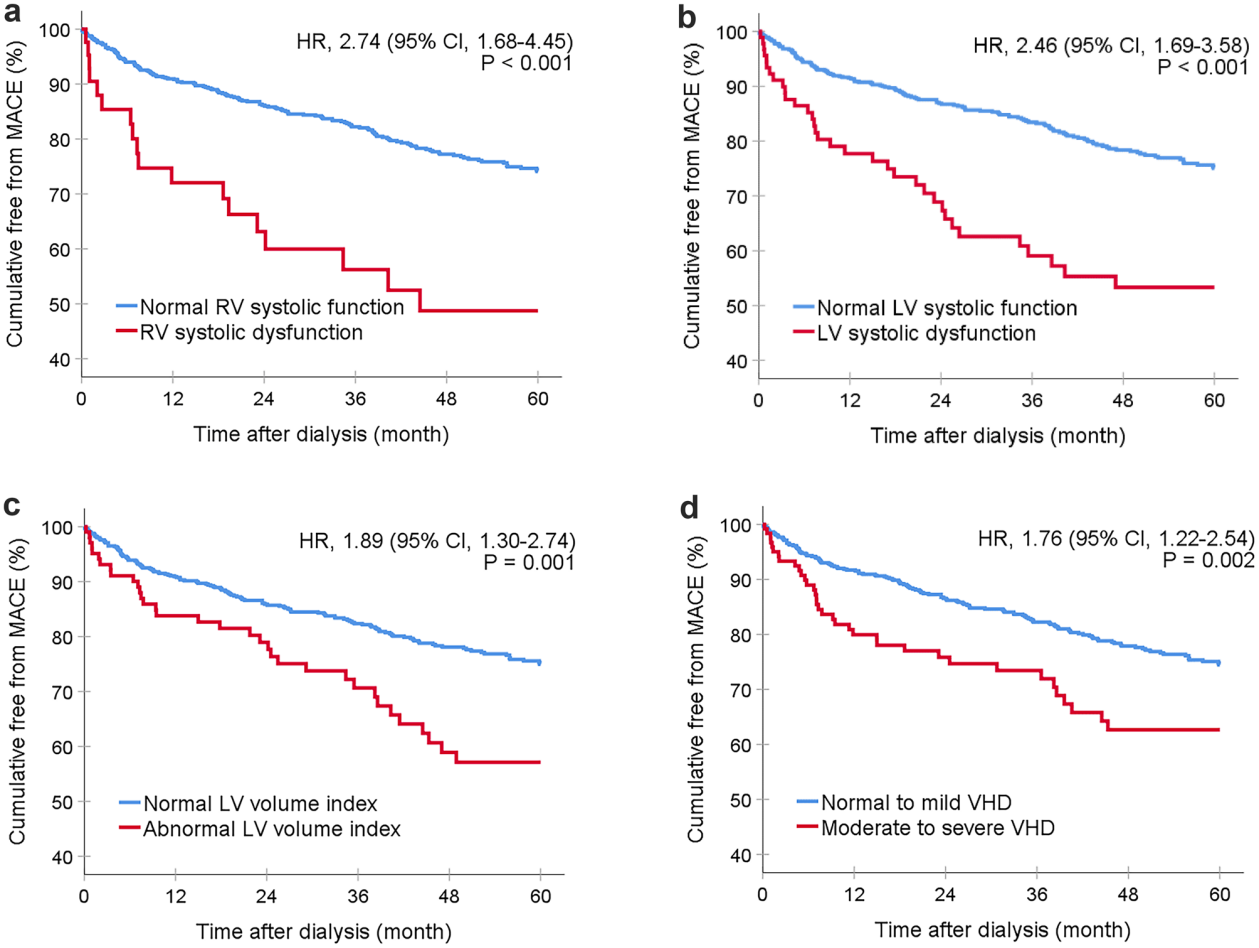
Impact of specific echocardiographic abnormalities on major adverse cardiovascular events (MACE) in dialysis patients. Kaplan–Meier survival curve analysis of mortality over a 5-year follow-up period in relation to specific echocardiographic abnormalities including right ventricular systolic dysfunction, left ventricular systolic dysfunction, left ventricular volume index, and moderate to severe valvular heart disease

Patients who exhibited abnormalities on echocardiography had significantly higher rates of both major adverse cardiovascular events (log-rank *p* = 0.002) and total mortality (log-rank *p* = 0.011) compared to those without abnormalities. The Kaplan–Meier figures demonstrated that all echocardiographic abnormalities, except for left ventricular hypertrophy and left atrial enlargement, were associated with an increased risk of 5-year all-cause mortality (Fig. 2). Additionally, most echocardiographic abnormalities, except for left ventricular hypertrophy, were found to be associated with an increased risk of major adverse cardiovascular events (Fig. 3). To assess the association between echocardiographic abnormalities and outcomes, we performed a Cox proportional hazard model analysis, adjusting for age, sex, comorbidities, and medications. Our findings showed that left ventricular systolic dysfunction (HR, 95% CI 2.31, 1.56–3.41, *p* < 0.001), left ventricular volume index (HR, 95% CI 1.93, 1.30–2.90, *p* = 0.001), right ventricular dysfunction (HR, 95% CI 1.86, 1.09–3.19, *p* = 0.024), valvular heart disease (HR, 95% CI 1.75, 1.22–2.51, *p* = 0.002), and pulmonary hypertension (HR, 95% CI 1.72, 1.25–2.34, *p* < 0.001) were significantly associated with a higher mortality rate (Table 3). Similarly, right ventricular systolic dysfunction (HR, 95% CI 2.36, 1.44–3.86, *p* = 0.001), left ventricular systolic dysfunction (HR, 95% CI 2.20, 1.47–3.30, *p* < 0.001), left ventricular volume index (HR, 95% CI 1.98, 1.34–2.94, *p* < 0.001), and valvular heart disease (HR, 95% CI 1.81, 1.25–2.61, *p* = 0.002) were identified as the independent risk factors for major adverse cardiovascular events. (Table 4).

**Table 3:** Risk of MACE in each echocardiographic abnormality

Echocardiographic abnormality variable	HR (95% CI)	*p* value
RV systolic dysfunction	2.36 (1.44–3.86)	0.001
Left ventricular ejection fraction	2.2 (1.47–3.30)	< 0.001
Left ventricular volume index	1.98 (1.34–2.94)	0.001
Valvular heart disease	1.81 (1.25–2.61)	0.002
Diastolic dysfunction	1.45 (1.07–1.97)	0.016
Pulmonary hypertension	1.30 (0.96–1.78)	0.094
Left ventricular hypertrophy	1.28 (0.94–1.74)	0.124
Left atrial enlargement	1.24 (0.91–1.68)	0.175

*Adjusted for age, gender, diabetes mellitus, ischemic stroke, atrial fibrillation, coronary artery disease, heart failure, and renin-angiotensin system

**Table 4:** Risk of 5-year all-cause mortality in each echocardiographic abnormality

Echocardiographic abnormality variable	HR (95% CI)	*p* value
Left ventricular ejection fraction	2.31 (1.56–3.41)	< 0.001
Left ventricular volume index	1.93 (1.30–2.90)	0.001
RV systolic dysfunction	1.86 (1.09–3.19)	0.024
Valvular heart disease	1.75 (1.22–2.51)	0.002
Pulmonary hypertension	1.72 (1.26–2.34)	< 0.001
Diastolic dysfunction	1.33 (0.98–1.79)	0.064
Left atrial enlargement	1.06 (0.79–1.43)	0.710
Left ventricular hypertrophy	0.97 (0.72–1.31)	0.820

*Adjusted for age, gender, diabetes mellitus, ischemic stroke, atrial fibrillation, coronary artery disease, heart failure, and renin-angiotensin system

### Subgroup analysis of the interaction between dialysis modalities and specific echocardiographic abnormalities

**Fig. 4:**
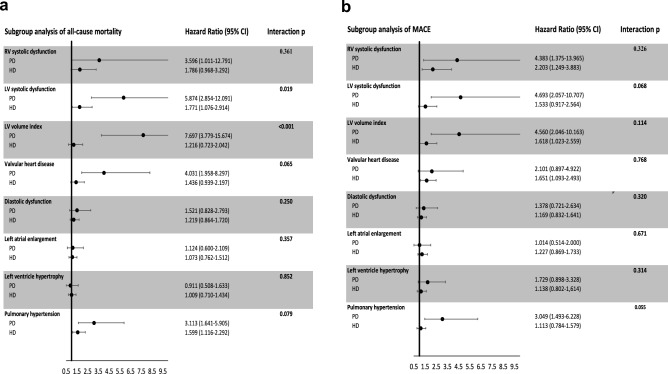
Association between distinct echocardiographic abnormalities and the risk of mortality and major adverse cardiovascular events (MACE) among individuals undergoing hemodialysis and peritoneal dialysis. Adjusted for age, gender, comorbidities, and medications. *HD* hemodialysis, *PD* peritoneal dialysis

The 5-year survival rate was 76.5% and 82.5% in HD and PD subjects, respectively. Hemodialysis can cause fluctuations in blood pressure and volume, as well as changes in electrolyte levels. In contrast, PD does not cause as many rapid shifts in fluid, but both HD and PD can have negative effects on the heart over time [17, 18]. To evaluate the interaction between dialysis modality and each echocardiographic abnormality on mortality and major adverse cardiovascular events, we performed subgroup analysis of interaction between dialysis modality and echocardiogram abnormalities on patient outcomes. The findings demonstrated that in PD patients, left ventricular systolic dysfunction (*p* for interaction = 0.019) and elevated left ventricular volume index (*p* for interaction < 0.001) were more strongly associated with increased mortality risk compared to HD patients (Fig. 4A). Conversely, there was no notable interaction between dialysis modality and echocardiographic abnormalities in predicting major adverse cardiovascular events (Fig. 4B).

## Discussion

The present study investigated the potential clinical implications of baseline echocardiography beyond its conventional use for assessing fluid status in kidney failure patients. Our findings demonstrated that a high percentage of patients exhibited at least one echocardiographic abnormality, with left ventricular hypertrophy and left atrial enlargement being the most common. However, these abnormalities were not found to be associated with adverse outcomes. On the other hand, left ventricular systolic dysfunction, diastolic dysfunction and valvular heart disease were found to be significant risk factors for major adverse cardiovascular events in kidney failure patients, consistent with their established association in the general population. In addition, right ventricular dysfunction and increased left ventricular volume index were also identified as predictors of poor outcome in this patient population. Furthermore, left ventricular systolic dysfunction and left ventricular volume index had a higher predictive value for mortality in patients on PD compared with patients on HD.

Although almost all echocardiographic parameters were associated with patient outcomes, left ventricular systolic function, right ventricular systolic function, and left ventricular volume index were the most important parameters associated with both major adverse cardiovascular events and mortality in our study. It is important to note that right ventricular systolic dysfunction was rare, occurring in only 5% of patients, but had the strongest association with major adverse cardiovascular events. In kidney failure patients, it is especially important to monitor right ventricular dysfunction due to the preload-dependent characteristic of the right ventricle and the vulnerability of fluid overload in this population [19]. In a study with 654 patients under chronic dialysis, Hickson et al., demonstrated that reduced left ventricular ejection fraction and right ventricular systolic dysfunction were independent risk factors for mortality [11]. However, our study, which had a larger sample size and longer follow-up period, revealed that abnormal left ventricular volume index and valvular heart disease were also independent risk factors for both major adverse cardiovascular events and death in this population.

Left ventricular hypertrophy and high left ventricular volume index represent distinct forms of left ventricular remodeling, each characterized by unique pathophysiological mechanisms. Left ventricular hypertrophy refers to an increase in mass of the left ventricle, which is commonly caused by chronic pressure overload. On the other hand, high left ventricular volume index refers to an enlargement of the left ventricular cavity, which is typically caused by chronic volume overload [20]. In the present study, left ventricular hypertrophy is the most prevalent echocardiographic abnormality in kidney failure patients. Left ventricular hypertrophy is a frequently encountered cardiac abnormality in kidney failure patients due to chronic hypertension; however, data from our study and other groups suggest left ventricular hypertrophy is not an independent factor of poor outcomes in dialysis patients [21–23]. In the present study, left atrial enlargement is also a prevalent echocardiography abnormality in kidney failure patients. Atrial fibrillation (Af), mitral valve disease, and left ventricular diastolic dysfunction are common etiologies for left atrial enlargement. Kidney failure patients have a higher prevalence of atrial fibrillation compared to the general population. Several studies have reported that the prevalence of atrial fibrillation in dialysis patients ranges from 8 to 27% [24, 25], which is significantly higher than the 1~2% prevalence of atrial fibrillation in the general population [26]. In the current study, 10% of the participants had mitral valve disease, and 49% had diastolic dysfunction. These findings were in line with the high occurrence of left atrial enlargement that reaches up to 45%. However, left atrial enlargement was not an independent predictor for either mortality or major adverse cardiovascular events.

Fluid overload is a common and important complication encountered in the care of kidney failure patients. Interestingly, we observed that the prognostic value of the baseline left ventricular systolic dysfunction and left ventricular volume index differed depending on the dialysis modality. Although left ventricular systolic dysfunction is a negative predictor of mortality in both PD and HD patients, its effect is more pronounced in PD patients than in HD patients (*p* for interaction = 0.019). Furthermore, higher left ventricular volume index is a significant risk factor for mortality in PD patients but not in HD patients (*p* for interaction < 0.001). A similar trend was also found in the prediction of major adverse cardiovascular events. Although there was no significant difference in the predictive value of pulmonary hypertension between PD and HD patients, there was a noticeable trend towards a higher predictive value in PD patients (with interaction *p* values of 0.079 for mortality and 0.055 for major adverse cardiovascular events. (Table 4) This lack of significance could be attributed to the broad standard deviation among PD patients, who were represented by a smaller sample size compared to HD patients (247 vs. 583, respectively). These observations highlight the challenge of fluid control in PD. Continuous and slower ultrafiltration rates during PD may increase the risk of fluid overload without causing any clinical symptoms [27, 28]. In a study of 311 patients, it was found that asymptomatic fluid overload is a frequent occurrence among incident PD patients and is strongly associated with both major adverse cardiovascular events and survival [29]. In contrast, HD patients require ultrafiltration to their preset dry weight thrice weekly and may face intradialytic hypotension due to excessive interdialytic weight gain, which imposes strict fluid intake restrictions [30, 31]. Furthermore, left ventricular systolic dysfunction and left ventricular dilatation may also contribute to fluid overload, which was previously mentioned as more common in PD patients and associated with poor outcomes. There is concern that removing too much fluid may result in residual renal function loss in PD [32]. However, it is worth mentioning that with cautious ultrafiltration, or by using objective measurements like bioimpedance, PD patients can reach their desired dry weight and blood pressure without compromising residual kidney function [33, 34].

There are several limitations to acknowledge in our study. Firstly, the study was conducted at a single center, which may limit the generalizability of the findings to other patient populations. Additionally, the study was retrospective in nature, which may introduce biases and confounding factors. Secondly, the echocardiographic measurements were performed by different operators, which may have resulted in some degree of variability in the results. Thirdly, echocardiography sessions were not uniformly timed with hemodialysis treatments. Nonetheless, echocardiography was conducted within three months of enrolling the participants. In our hospital, establishing a new patient’s dry weight generally takes about one month, encompassing 10–13 dialysis sessions. This process, while specific to our institution, acknowledges the diversity in clinical protocols across various healthcare environments. Consequently, the participants were predominantly stable and not experiencing decompensated states, with most achieving their dry weight by the time of the echocardiography. This approach likely reduced the impact of fluid status on our findings, helping to mitigate potential bias. Fourthly, left atrial size, but not left atrial volume index, was used as an indicator of diastolic dysfunction. This is because the study population was recruited before the publication of the latest left ventricular diastolic dysfunction guideline and the limitation of the echocardiographic machine [15]. Lastly, although the study identified left ventricular systolic function, right ventricular systolic function, and left ventricular volume index as important predictors of major adverse cardiovascular events and mortality, further investigation is needed to understand the complex interplay between these three cardiac abnormalities as predictors of major adverse cardiovascular events and mortality.

In conclusion, this study provides insights into echocardiographic abnormalities and their association with adverse outcomes in kidney failure patients, which can help clinicians optimize the management of patients and improve their overall health. The present study describes a high prevalence of echocardiographic abnormalities in kidney failure patients, with left ventricular systolic function, right ventricular systolic function, and left ventricular volume index being the most important predictors of both major adverse cardiovascular events and mortality. Although left ventricular hypertrophy and left atrial enlargement were the most common abnormalities, they were not associated with adverse outcomes. The prognostic value of the baseline left ventricular volume index and left ventricular systolic dysfunction differed depending on the dialysis modality, being less critical in HD compared to PD.

## Supplementary Information

Below is the link to the electronic supplementary material.

Supplementary file1 (DOCX 19 KB)

## Data Availability

The data supporting the findings of this study are available within the article and its supplementary materials.
