# Supplementary material for: Beyond prevalence: significance and differential impact of echocardiographic abnormalities in dialysis patients
Source: J Nephrol. 2024 Jun 4;37(5):1261–71. doi: 10.1007/s40620-024-01963-2 (PMC11405503; doi:10.1007/s40620-024-01963-2)
Supplement: Supplementary file 1 — Supplementary file1 (DOCX 19 KB) [file 40620_2024_1963_MOESM1_ESM.docx]

**Supplementary table 1**. Echocardiography parameters on different vascular access

|  | **AVF**  **(n = 244)** | **CVC**  **(n = 339)** | ***P* value** |
| --- | --- | --- | --- |
| ***Echocardiographic parameters*** |  |  |  |
| LV mass index, g/m2 | 129.18 ± 41.19 | 123.38 ± 38.86 | 0.053 |
| LV volume index, ml/m2 | 51.57 ± 17.39 | 50.77 ±18.67 | 0.555 |
| LVEF, % | 56.03 ± 9.03 | 55.69 ± 9.26 | 0.624 |
| LA dimension | 41.49 ± 7.15 | 39.80 ± 7.66 | 0.003 |
| Med e’, cm/s | 5.52 ± 1.54 | 5.47 ± 1.61 | 0.708 |
| Med E/e’ | 17.02 ± 7.63 | 16.67 ± 6.91 | 0.518 |
| PASP, mmHg | 37.18 ± 14.94 | 35.03 ± 14.71 | 0.064 |
| RV systolic dysfunction, % | 15 (6.1) | 27 (4.4) | 0.286 |
| AVF, arteriatovenous fistula; CVC, central venous catheter; LA, left atrium; LV, left ventricle; LVEF, left ventricular ejection fraction; LVMi, left ventricular mass index; PASP, pulmonary artery systolic pressure; RV, right ventricle; TR, tricuspid regurgitation. | | | |

| **Table 2. Univariate cox proportional regression analysis of predictors of 5-year mortality, using cox proportional regression analysis** | | |
| --- | --- | --- |
|  | **Univariate** | |
| **Variables** | **HR (95% CI)** | **P value** |
| Age | 1.04 (1.03-1.05) | < 0.01 |
| Sex | 1.40 (1.05-1.88) | 0.02 |
| **Comorbidities** |  |  |
| Hypertension | 0.88 (0.62-1.27) | 0.50 |
| Diabetes mellitus | 1.51 (1.13-2.02) | < 0.01 |
| Ischemic stroke | 2.22 (1.43-3.43) | < 0.01 |
| Atrial fibrillation | 2.47 (1.68-3.63) | < 0.01 |
| Coronary artery disease | 1.95 (1.42-2.69) | < 0.01 |
| Heart failure | 1.90 (1.35-2.67) | < 0.01 |
| **Medication** |  |  |
| Beta-blocker | 0.82 (0.61-1.10) | 0.19 |
| RAS inhibitor | 0.73 (0.53-0.99) | 0.04 |
| CI: confidence interval; HR: hazard ratios; RAS: Renin-angiotensin system | | |
